# Supplementary material for: Patterns of Intron Gain and Loss in Fungi
Source: PLoS Biol. 2004 Nov 30;2(12):e422. doi: 10.1371/journal.pbio.0020422 (PMC532390; doi:10.1371/journal.pbio.0020422)
Supplement: Table S1 — Also available at http://genes.mit.edu/NielsenEtAl/. (4.3 MB ZIP). [file pbio.0020422.st001.zip › NielsenEtAl/html/890.html]

AN4687.1.NCU02127.1.MG02541.1.FG08509.1


```
 CLUSTAL W (1.82) Multiple Sequence Alignments - Introns Inserted


Sequence 1: MG02541.1	605 aa
Sequence 2: FG08509.1	571 aa
Sequence 3: NCU02127.1	728 aa
Sequence 4: AN4687.1	633 aa
Alignment Length: 732 aa
Number Identitical Residues: 357 aa
Alignment Score (without introns) 15986


MG02541.1 	MSLTRPSR-EFLHLPRQLARTTSRTRPSSTGSNTGITTNQTRRISTLPQTRPSRQTAAPT
NCU02127.1	MSFSSASRREVLLFSKQLSRAASTSPRSASLPLHPRNLGLLLHSNQQQPQQSQRQQSLLQ
FG08509.1 	MALSRSSR-QAMLLGRQFGRQSSRLQISS-------------------------------
AN4687.1  	MRVSVPLHSIRRSCNSSIANAATRLTPQRLYLSYG------------------RRSSIVQ
          	* .: . :        .:.. ::    .                            :   

MG02541.1 	QTPIRQTRSVATFTPPFQAGAVSRIETNVDPTSPEFRENEASMAELMTRLDTLTRKAQQG
NCU02127.1	Q----QRRSVATFTTPHQANAISVLKSDVDTDSPEFKENERLMGEAMARLEELTRKVHQG
FG08509.1 	-----QRRAVANLTPPHHAGAISRIQTSVDPSSEEFKENEKQMSEVMSRMQELARKIQKG
AN4687.1  	AHPRTQARPIASYTHPHHASAISVLSTAVDTSSPDFRENERQMQEVLNRMNSLHSTISQG
          	  .  * *.:*. * *.:*.*:* :.: **. * :*:***  * * : *:: *  .  :*

MG02541.1 	GNAKAKEKHIARGKMLPRDRIAALIDPGSSFMELSALAAHEVYPEADVPAAGIITGVGVV
NCU02127.1	GPQKAREKHLLRKKMLPRDRITALIDPGTTFLELSPLAGHELYPEADVPAGGIITGVGVV
FG08509.1 	GSDKARQKHIDRKKMLPRDRVTALIDPGTTFMELSPMAGYDLYPEAEVPAGGIITGVGVV
AN4687.1  	GPQKAKDKHVARGKMLPRDRVSALIDPGTSFLELSQLAGHEVYPGEDVPAGGIITGIGTV
          	*  **::**: * *******::******::*:*** :*.:::**  :***.*****:*.*

MG02541.1 	EGVTCMIVANDSTVKGGTYYPMTVKKHLRAQAVAQENRLP2CIYLVDSGGANLPHQADVF
NCU02127.1	EGVTCVIVANDSTVKGGTYYPITVKKHLRAQEVAKENNLP2CIYLVDSGGANLPHQSDVF
FG08509.1 	EGVTCVIVANDSTVKGGTYYPITVKKHLRAQAVARENKLP2CIYLVDSGGANLPHQADVF
AN4687.1  	EGVTCMIVANDSTVKGGTYYPVTVKKHLRAQAIAQENKLP~CIYLVDSGGANLPHQADVF
          	*****:***************:********* :*:**.** ***************:***

MG02541.1 	PDQNHFGRIFYNQARMSSQGIPQIAVVMGPCTAGGAYVPAMSDESIIVGNQGHIFLAGPP
NCU02127.1	PDREHFGRIFFNQARMSSQGIPQIAVVMGPCTAGGAYVPAMSDESIIVQEQGHIFLAGPP
FG08509.1 	PDQNHFGRIFYNQARMSSEGIPQIAVVMGPCTAGGAYVPAMSDESIIVQEQGHIFLAGPP
AN4687.1  	PDKEHFGRIFFNQARMSSQGIPQISVVMGPCTAGGAYVPAMSDETIIVENQGTIFLAGPP
          	**::******:*******:*****:*******************:*** :** *******

MG02541.1 	LVKAATGEVVTPEELGGGEMHSSVSGVTDYLAVDDAHALVLARRSVSNLNWPTNESK---
NCU02127.1	LVKAATGEVVSPEELGGGAMHSSVSGVTDYLAVDDAHAIVLARRSIANLNWPRGSSTGAV
FG08509.1 	LVKAATGEVVSHEDLGGGKMHSSVSGVTDYLAVDDAHAVVLARRCISNLNWPKKSPE---
AN4687.1  	LVKAATGEVVSAEDLGGGQLHSTISGVTDYLAVDDAHAIVLARRSISNLNYPKAKQP---
          	**********: *:**** :**::**************:*****.::***:*  .     

MG02541.1 	-SSQ-ELSYEEPLYPAEELLGIASPNLRKPLPIREVIARIVDGSAFSEFKRDYGTTLVTG
NCU02127.1	TPADFEQKFEEPLYSADELLGIASTNLRKPLPIHEVIARIVDGSAFAEFKRDYGTTLVTG
FG08509.1 	-TQAPKPTYLEPLHNPEELLGIATTNLRKPLPIREVIARVVDGSEFSEFKRDFGTTLVTG
AN4687.1  	--LESNDDIKEPLYDPAELNGIVGTNLRRQIPVHEVIARIVDGSEFAEFKRDYGTTLVTG
          	     :    ***: . ** **. .***: :*::*****:**** *:*****:*******

MG02541.1 	FASIYGQKVGIVANDGVLLGTSAVKGAHFIELCAQRGVPLVFLQNISGFMVGKEAERDGI
NCU02127.1	FAKIYGHRVGIVANNGILFSSSALKGAHFIELCAQRGIPLVFLQNISGFMVGRDAEREGI
FG08509.1 	FAEIYGHKVGIVANDGILFAKSAVKGAHFIELCSQRGIPLVFLQNISGFMVGSESEREGI
AN4687.1  	FARIHGHRVGIVANNGILFSESSLKGAHFIELCAQRNIPLVFLQNISGFMVGADAEKGGI
          	** *:*::******:*:*:. *::*********:**.:************** ::*: **

MG02541.1 	AKHGAKLVTAVACADVPKFTVVVGGSYGAGNYGMC~GRAYSPRFMWLWPNARVGVMGAEQ
NCU02127.1	AKNGAKLVTAVACADVPKFTVVVGGSYGAGNYGMC~GRAYSPRFLWMWPNARIGVMGSEQ
FG08509.1 	AKHGAKLVTAVACADVPKFTVVIGGSYGAGNYGMC~GRAYSPRFLWMWPNAKVGVMGSEQ
AN4687.1  	AKNGAKLVTAVACADVPKFTVVFGSSAGAGNYGMC2GRAYSPRFLFMWPNAKIGVMGSEQ
          	**:*******************.*.* ******** ********:::****::****:**

MG02541.1 	LAAVMETVGK-PDPELKDRIEKETDAVFSSARLW0DDGVIPPQHTRRYLGLGLRAAMSG~
NCU02127.1	LAKVMETVGTKADPELQDRIERESDAVFSSARLW~DDGVIPPQHTRRYLGLGLRAALGG~
FG08509.1 	LAAVMETVGKTVDAGLKERIEKESDATFSSARLW0DDGIIPPQHTRRYLGLGLQAAMGG~
AN4687.1  	LSSVMEAVGRTADPELKARIDRESEATFSSARLW~DDGIIPPAHTRHVLGLSLAAALGV1
          	*: ***:** . *. *: **::*::*.******* ***:*** ***: ***.* **:.  

MG02541.1 	RNEVKPG~NTKFGVFRM~------------------------------------------
NCU02127.1	RNEQAPR~GTKFGVFRI2FILLCLLRGELYRGNGDLTYSTSPSSTEPLAPRRISTMPPSR
FG08509.1 	RNEVKAG~DTKFGVFRM~------------------------------------------
AN4687.1  	CIITISC1HRATMTIKK~-----------------------------EETPYNGWMISGQ
          	     .       .::                                 .   .   .. 

MG02541.1 	---------------------~--------------------------------------
NCU02127.1	FRQKVRGNGELDEKVESLRAG2SRSTKQNREKANSLNNWQVLEAKSEDPLRSAGVLKHPS
FG08509.1 	---------------------~--------------------------------------
AN4687.1  	FKNLLAGDHEASMDETTERTQ~CYIRYKSSHY----------------------------
          	  .   .. . . .  :  :  .    ..                               

MG02541.1 	-------------------
NCU02127.1	PALDIIQKYIPAGLVCLSA
FG08509.1 	-------------------
AN4687.1  	-------------------
          	
```
